# Predicting Sepsis in Heart Failure Patients Supported by Left Ventricular Assist Devices: The Role of VE-Cadherin and ADAM10

**DOI:** 10.3390/ijms27020563

**Published:** 2026-01-06

**Authors:** Shiyi Li, Iván Murrieta-Álvarez, Ismael Garcia, Katherine V. Nordick, Rishav Bhattacharya, Shreyo Ghosh, Ronald A. Shaju, Adel M. Hassan, Carl P. Walther, Camila Hochman-Mendez, Alexis E. Shafii, Kenneth K. Liao, Nandan K. Mondal

**Affiliations:** 1 Zhongnan Hospital of Wuhan University, Wuhan University, Wuhan 430071, China; 2 Michael E. DeBakey Department of Surgery, Division of Cardiothoracic Transplantation and Circulatory Support, Baylor College of Medicine, Houston, TX 77030, USA; 3 Selzman Institute for Kidney Health, Section of Nephrology, Department of Medicine, Baylor College of Medicine, Houston, TX 77030, USA; 4 Department of Regenerative Medicine Research, Texas Heart Institute, Houston, TX 77030, USA

**Keywords:** mechanical circulatory support device, sepsis, VE-Cadherin, ADAM10, vascular permeability

## Abstract

Vascular endothelial cadherin (VE-Cadherin) is a major endothelial adhesion molecule and can be cleaved explicitly by metalloproteinase domain-containing protein 10 (ADAM10). Vascular hyperpermeability may contribute to a greater susceptibility to sepsis in left ventricular assist device (LVAD) support patients. We aim to evaluate the efficacy of VE-Cadherin and ADAM10 for predicting sepsis in LVAD patients. We prospectively recruited 50 patients with advanced heart failure receiving LVAD therapy. Baseline and weekly postoperative blood samples (weeks 1–4) were collected, and plasma VE-cadherin and ADAM10 levels were measured. Sepsis occurred in 9 of 50 patients (18.0%). Across all sampling points, plasma VE-cadherin and ADAM10 levels were significantly higher in the sepsis group relative to the non-sepsis group. From pre-implantation to 1-week and 1-month post-operation, VE-Cadherin alone showed good performance for sepsis prediction, with areas under the receiver operating characteristic (AUC) of 0.75, 0.81, 0.69, 0.72, and 0.77, respectively. A significant positive correlation between VE-cadherin and ADAM10 was detected only among sepsis patients. Incorporating ADAM10 into the prediction models significantly enhances their predictive performance. Plasma VE-Cadherin levels can be a valuable biomarker for predicting sepsis in LVAD patients, with predictive performance further enhanced when combined with circulating ADAM10 levels.

## 1. Introduction

Despite heart transplantation remaining the “gold standard” therapy for end-stage heart failure (HF) patients, a shortage of available donor hearts is the main factor limiting access to transplantation. Left ventricular assist device (LVAD) has emerged as an effective alternative treatment for end-stage HF, either as a bridge to transplantation (BTT) or as destination therapy (DT) [1,2,3]. With significant advances in mechanical circulatory support technology over the last decade, survival after LVAD implantation has improved [4]. However, infection remains one of the major concerns for LVAD patients, especially for sepsis, which can contribute to high morbidity and mortality [5,6]. Unlike the general population, LVAD patients exhibit impaired immune function [7,8]. Additionally, the presence of an external driveline increases susceptibility to infection [9], which may progress to sepsis. Therefore, it is crucial to identify a reliable biomarker that can early identify high-risk patients, enabling physicians to implement personalized management strategies, including timely, more aggressive interventions to prevent the onset or progression of sepsis.

Sepsis is defined as a dysregulated host response to infection that results in life-threatening organ dysfunction [10,11]. Published data have consistently shown that disruption of the endothelial barrier and increased endothelial hyperpermeability are the hallmarks in the pathogenesis of sepsis and systemic inflammation [12,13,14]. The endothelial barrier is maintained by tight and adherens junctions, with vascular endothelial (VE)-cadherin serving as the major transmembrane adhesion molecule within adherens junctions and a key determinant of endothelial barrier integrity [15]. Disruption of VE-cadherin will make it easier for pathogens to penetrate the endothelial barrier, inducing a cytokine burst that eventually progresses to sepsis [16]. Moreover, loss of VE-cadherin integrity promotes endothelial junction disassembly, resulting in increased vascular leakage, tissue edema, and fluid accumulation that can progress to multiorgan failure and adverse outcomes [17,18]. Previous studies revealed that an elevated level of circulating VE-cadherin is associated with sepsis severity [18,19]. VE-cadherin has been proposed as a promising biomarker to classify sepsis in different settings [20,21]. However, these studies mainly focused on VE-cadherin’s predictive ability for sepsis classification and prognosis. The predictive value of VE-cadherin for sepsis remains unclear.

The shedding of VE-cadherin is mainly mediated by the family of zinc-dependent transmembrane disintegrin metalloproteinases (ADAMs). Among them, ADAM10 functions as a “molecular scissor,” facilitating VE-Cadherin cleavage and shedding [22,23], which contributes to increased endothelial permeability [24]. In addition, ADAM10 influences the production of inflammatory cytokines; its excessive activation has been linked to systemic inflammatory responses and disruption of the vascular endothelial barrier following sepsis [21,25,26]. A recent study has identified ADAM10 as essential for the pathogenesis of Staphylococcus sepsis [27]. Staphylococcus species are the most common pathogens causing sepsis in LVAD patients [28]. ADAM10 may play a crucial role in the development of sepsis in LVAD patients.

We conducted a 3.5-year prospective, single-center observational cohort study to investigate plasma VE-cadherin levels as a predictor of sepsis within the first 180 days after LVAD implantation.

## 2. Results

### 2.1. Total Patient Characteristics

**Figure 1:**
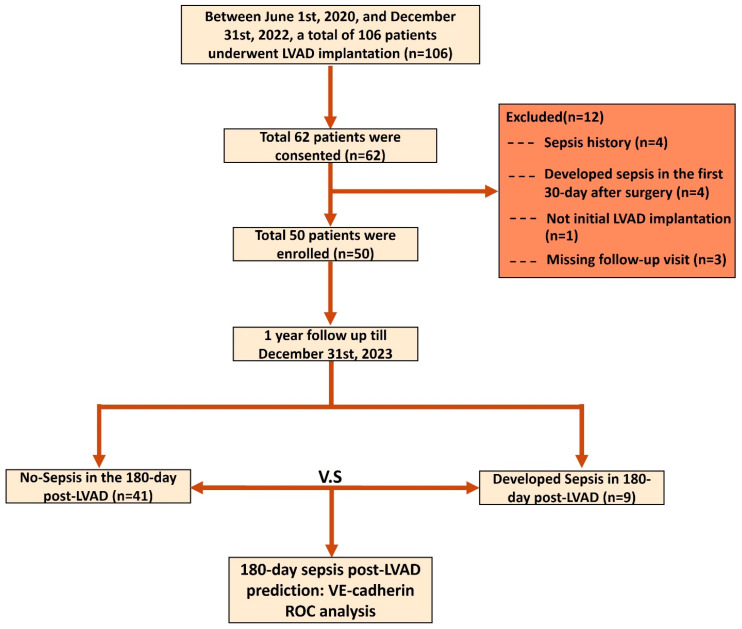
Flowchart illustrating patient eligibility assessment, cohort grouping, and subsequent analyses. LVAD, left ventricular assist device; ROC, receiver operating characteristic curve; VE-Cadherin, vascular endothelial-cadherin.

From 1 June 2020, to 31 December 2022, a total of 106 patients with advanced heart failure were scheduled for LVAD implantation at our institution. Of these, 62 patients provided consent to participate in the study. After excluding patients with prior sepsis history (*n* = 4), sepsis within the first 30 postoperative days (*n* = 4), non-initial LVAD implantation (*n* = 1), and missing follow-up (*n* = 3), 50 patients were enrolled and included in the final analysis. During the 180-day postoperative follow-up period, nine patients (18.0%) developed sepsis. The patient screening, exclusion criteria, and cohort enrollment process are summarized in Figure 1.

### 2.2. Characteristics of Patients with Sepsis and Those Without Sepsis

The demographic and clinical characteristics of the sepsis and no-sepsis groups are outlined in Table 1. Nine patients (18%) experienced sepsis after LVAD implantation, while forty-one patients (82%) did not experience sepsis during the follow-up visit period. The majority of patients are male, Caucasian, and white in both the sepsis and the no-sepsis groups. Over half of the patients in both the sepsis and non-sepsis groups had ischemic cardiomyopathy with a left ventricular ejection fraction < 20% at presentation. The population predominantly consisted of New York Heart Association (NYHA) class IV patients with a median Interagency Registry for Mechanically Assisted Circulatory Support (INTERMACS) profile of 2.3. Most patients received a HeartMate III LVAD, and LVAD implantation was intended as destination therapy in both the no-sepsis and sepsis groups. Four patients (44.44%) died in the sepsis group, while all patients survived in the no-sepsis group within the first year after LVAD implantation.

A total of nine patients (18.0%) developed sepsis between post-operative day 30 and post-operative day 180, with a median onset at day 56 (Median day is 56, range from 33 to 127). Among the nine patients who developed sepsis, four met the Sepsis-3 criteria for septic shock, requiring vasopressor therapy to maintain hemodynamic stability and demonstrating elevated lactate levels during the acute episode. An additional three patients exhibited lactate elevation during sepsis but did not require vasopressor support. The remaining two patients had organ dysfunction consistent with sepsis, but neither had a vasopressor requirement nor lactate elevation. Regarding respiratory involvement, three sepsis patients developed acute respiratory distress syndrome (ARDS) and required Extracorporeal Membrane Oxygenation (ECMO) support. One additional patient required ECMO due to cardiogenic shock. Three other sepsis patients required mechanical ventilation without ECMO, whereas two patients experienced milder courses of sepsis without the need for mechanical ventilation. Among the identified infections, more than half were non-Mechanical Circulatory Support (MCS)-specific. This included three cases of bacteremia originating from urinary tract infections and two cases of pneumonia. Approximately 40% of infections were specific to MCS, including one case of device-related endocarditis and three cases of device-related bloodstream infections, originating either from the percutaneous driveline exit site or the pump pocket. Additionally, bacterial pathogens were the predominant cause of sepsis, while only a small proportion of patients developed sepsis due to fungal infections.

### 2.3. Comparison of Hematologic and Blood Chemistry Profiles Between Sepsis and No-Sepsis Patients

**Figure 2:**
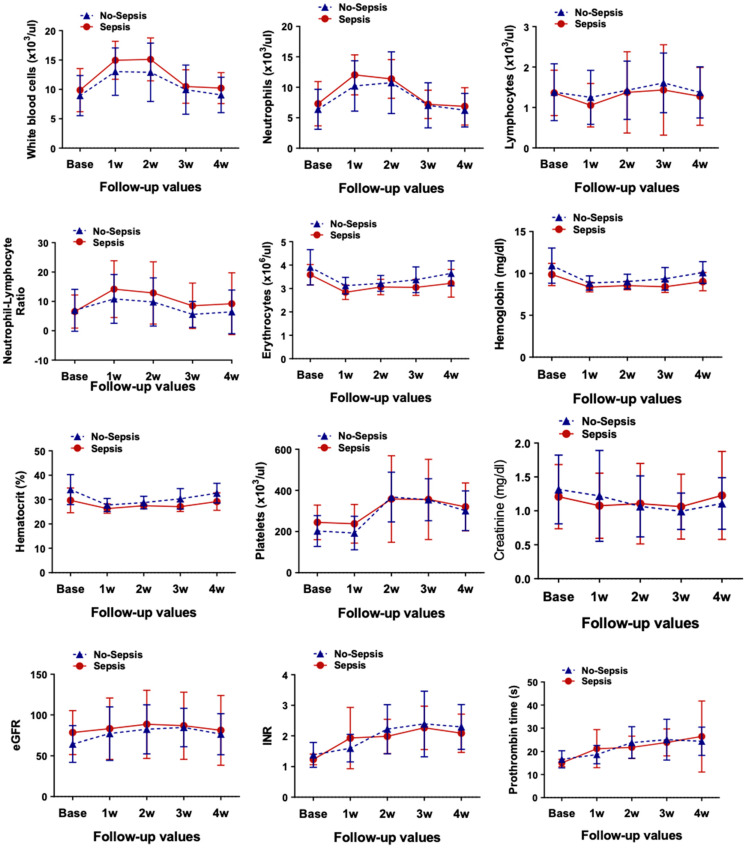
Time-dependent variations in standard hematology and blood chemistry values at baseline and during follow-up after LVAD implantation in the sepsis and non-sepsis groups. Data are expressed as mean ± standard deviation (SD), *p* < 0.05 is considered significant. eGFR, estimated glomerular filtration rate; INR, international normalized ratio.

When evaluating hematologic and blood chemistry parameters between sepsis and no-sepsis patients during the 1–6-month postoperative period, all parameters were comparable between the two groups. Total white blood cell (WBC) counts and WBC differentials, including neutrophils, lymphocytes, neutrophil/lymphocyte ratios, and monocytes, were comparable between groups at all time points. Moreover, erythrocyte count, hemoglobin level, and platelet count were all comparable before and after LVAD implantation in the first month. The lab profiles between patients with and without sepsis are presented in Figure 2.

### 2.4. Trajectories of ADAM10 and VE-Cadherin in HF Patients Before and After LVAD Implantation and Their Relationship

**Figure 3:**
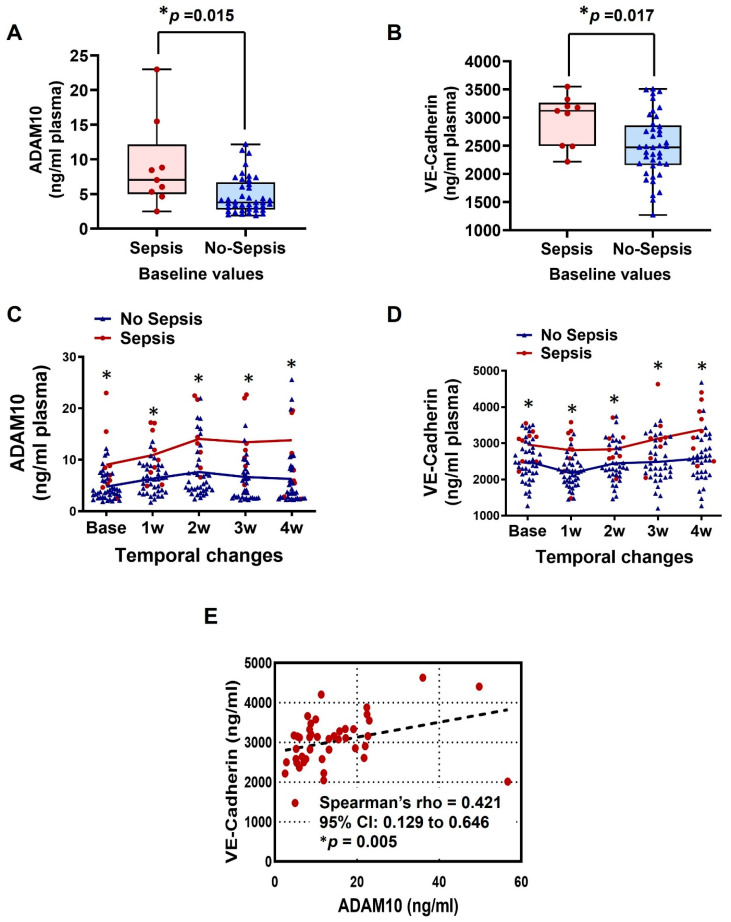
Box-and-whisker plots of baseline measurements demonstrate differences in plasma ADAM10 (**A**) and VE-cadherin (**B**) levels between patients who later developed sepsis and those who did not. Horizontal lines within each box denote the median; whiskers indicate the minimum and maximum values within the confidence limits. Line plots depict longitudinal changes in plasma ADAM10 (**C**) and VE-cadherin (**D**) from pre-implantation through postoperative time points in the sepsis and non-sepsis groups. Panel (**E**) illustrates the Spearman correlation between ADAM10 and VE-cadherin among patients with sepsis. Data are presented as median with SD. * *p* < 0.05 is considered significant. VE-Cadherin, vascular endothelial-cadherin; ADAM10, disintegrin metalloproteinases 10.

Baseline elevated plasma ADAM10 and VE-Cadherin levels were observed in the sepsis group patients when compared to the no-sepsis group patients (Figure 3A,B). Moreover, after LVAD implantation, patients in the sepsis group had significantly higher plasma levels of ADAM10 and VE-Cadherin than those without sepsis (Figure 3C,D). Furthermore, we observed that circulating ADAM10 levels increased dramatically within the first 2 weeks, then returned to near baseline levels in the no-sepsis group, whereas they remained elevated in the sepsis group (Figure 3C). For circulating VE-Cadherin, both groups showed decreases in the first week after LVAD implantation, followed by a return to baseline levels in the no-sepsis group, whereas it gradually increased in the sepsis group (Figure 3D). Spearman correlation analysis revealed a significant positive association between ADAM10 and VE-Cadherin in sepsis patients (rho = 0.421, *p* = 0.005; Figure 3E).

To evaluate group and time effects on biomarker levels, a two-way analysis of variance (ANOVA) with post hoc testing was performed. The results showed that both group and time were significant main effects for ADAM10 and VE-Cadherin, with no significant group-by-time interaction for either marker (Table 2).

### 2.5. VE-Cadherin Can Be a Reliable Biomarker for Predicting Sepsis in LVAD Patients

**Figure 4:**
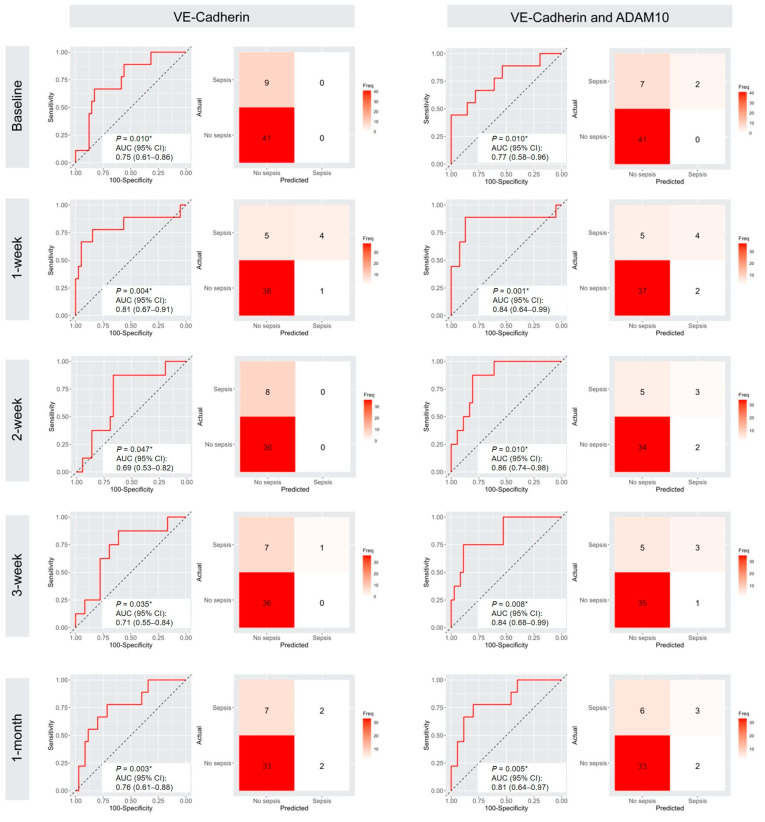
ROC curves as well as confusion matrices of the multi-timepoints value (Baseline, 1 week, 2 week, 3 week, and 1 month) of circulating VE-Cadherin and VE-Cadherin combined with ADAM10 for predicting sepsis after LVAD insertion. ROC, receiver operating characteristic; AUC, area under the ROC. VE-Cadherin, vascular endothelial-cadherin; ADAM10, disintegrin metalloproteinases 10. * *p* < 0.05 is considered significant.

We consistently observed significantly higher plasma VE-Cadherin levels before and after LVAD implantation in patients with sepsis. To evaluate its predictive value for postoperative sepsis in LVAD patients, we analyzed VE-Cadherin levels across multiple time points. Baseline plasma VE-Cadherin demonstrated an area under the curve (AUC) of 0.75 (95% confidence interval (CI): 0.611–0.864, *p* = 0.010), with a sensitivity of 66.7% and specificity of 82.9%. VE-Cadherin exhibited the highest predictive performance at postoperative week 1, with an AUC of 0.81 (95% CI: 0.676–0.912, *p* = 0.004), sensitivity of 77.8%, and specificity of 84.6%. At postoperative week 2, the AUC declined to 0.69 (95% CI: 0.537–0.824; *p* = 0.047), with sensitivities and specificities of 87.5% and 66.7%, respectively. At postoperative week 3, VE-Cadherin levels yielded an AUC of 0.71 (95% CI: 0.559–0.841, *p* = 0.035), with a sensitivity of 87.5% and a specificity of 61.1%. By postoperative week 4, VE-Cadherin demonstrated an AUC of 0.76 (95% CI: 0.617–0.882, *p* = 0.003), with 77.8% sensitivity and 71.4% specificity. We also observed significantly higher plasma ADAM10 levels across all time points and a strong positive correlation between circulating ADAM10 and VE-Cadherin, observed exclusively in patients with sepsis. Further analysis incorporating ADAM10 with VE-Cadherin yielded prediction models with greatly improved predictive performance across all time points. At baseline, it showed an AUC of 0.77 (95% CI: 0.58–0.96, *p* = 0.010). At week 1 after implantation, the AUC improved to 0.84 (95% CI: 0.64–0.99, *p* = 0.001). At weeks 2 and 3, it yielded AUCs of 0.86 (95% CI: 0.74–0.98; *p* = 0.010) and 0.84 (95% CI: 0.68–0.99; *p* = 0.008), respectively. After the first month of implantation, the combined model showed an AUC of 0.81 (95% CI: 0.64–0.97, *p* = 0.005). The predictive performance of VE-Cadherin and VE-Cadherin combined with ADAM10 for post-operative sepsis is shown in Table 3, and their ROC curves are shown in Figure 4.

**Figure 5:**
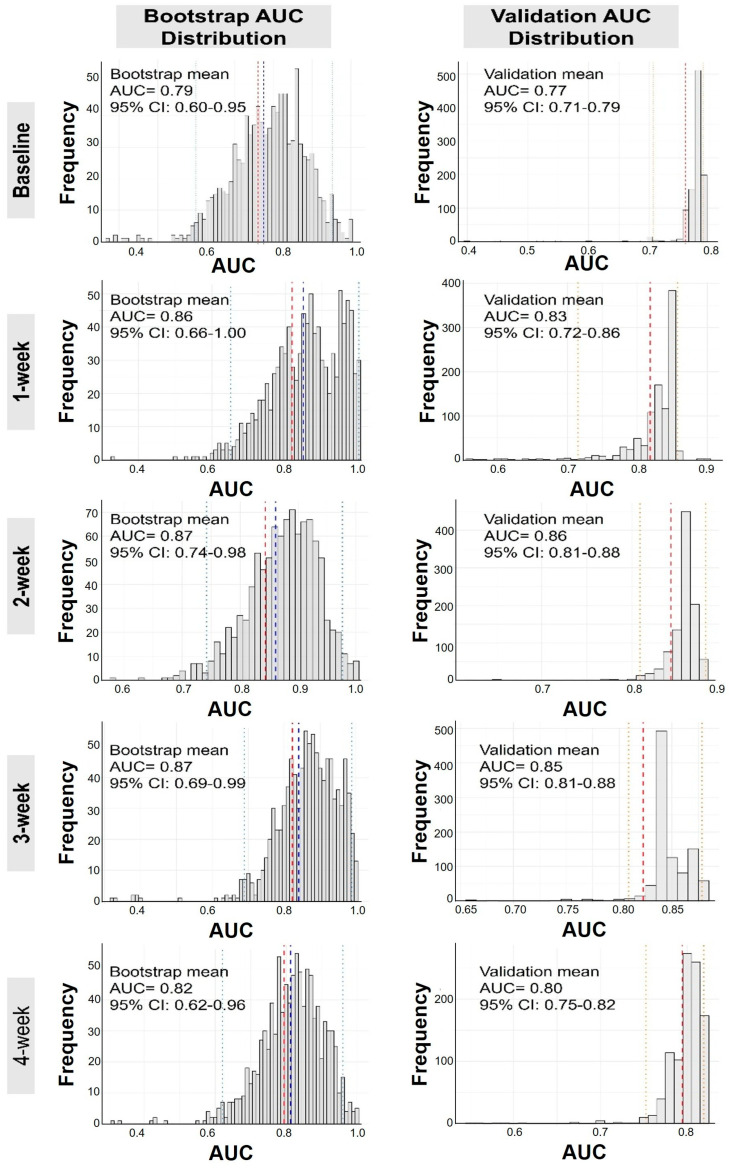
Histogram distributions of AUC values from 1000 bootstrap resampling and validation analyses across multiple timepoints (Baseline, 1 week, 2 week, 3 week, and 1 month) for circulating VE-Cadherin and ADAM10 combined models in predicting sepsis. The blue line represents the apparent AUC, the red line indicates the optimism-corrected AUC (adjusted for potential overfitting), and the light dotted lines denote the 95% confidence intervals (CI). AUC, area under the ROC curve; CI, confidence interval.

Due to the small sample size and the number of events, we performed bootstrap resampling and validation analyses [29]. From baseline to post-operative month 1, the bootstrap resampling showed the average AUC of 0.79 (95% CI: 0.60–0.96) at baseline; 0.86 (95% CI: 0.65–1.00) at post-operative week 1; 0.87 (95% CI: 0.74–0.98) at post-operative week 2; 0.87 (95% CI: 0.69–0.99) at post-operative week 3 and 0.82 (95% CI: 0.62–0.96) at post-operative month 1; the validation analysis yielded AUC of 0.77 (95% CI: 0.71–0.79) at baseline; 0.83 (95% CI: 0.72–0.86) at post-operative week 1; 0.86 (95% CI: 0.81–0.88) at post-operative week 2; 0.85 (95% CI: 0.81–0.88) at post-operative week 3; 0.80 (95% CI: 0.75–0.82) at post-operative week 4. The distribution of AUCs from 1000 bootstrap resamples and validation analyses is presented as histograms in Figure 5.

## 3. Discussion

The MOMENTUM 3 trial, which studied MagLev technology in 1020 patients using the HeartMate 3 device, found that infection is the most common major adverse event in LVAD patients; sepsis notably increases the risk of mortality, with a hazard ratio of 2.57 compared with patients without sepsis [30]. Moreover, Sakir et al. used univariable and multivariable analyses, as well as regression analysis, to identify risk factors for early mortality after LVAD implantation. They found that sepsis and multi-organ failure are the leading causes of early mortality in advanced heart failure patients after receiving LVAD implantation therapy [31]. Consistent with prior studies, we found that sepsis patients had significantly higher 1-year mortality than patients in the no-sepsis group. In our single-center study, four patients died, representing 44.44% of the sepsis group patients in the first year after LVAD implantation. Sepsis is a severe and life-threatening complication following LVAD implantation, significantly increasing postoperative mortality. Therefore, a valuable and reliable biomarker that enables physicians to detect sepsis risk early in LVAD patients is critical for optimizing postoperative management. In this study, we evaluated the predictive performance of VE-Cadherin at multiple time points (before and after LVAD implantation) for post-operative sepsis prediction. We found that VE-Cadherin may serve as a valuable and reliable biomarker for predicting postoperative sepsis in LVAD populations. Furthermore, combining VE-Cadherin with ADAM10 levels markedly enhanced its predictive accuracy.

VE-Cadherin is the principal cell–cell adhesion molecule of the endothelial adherent junction in the control of vascular permeability [32]. Increased shedding of VE-Cadherin can lead to vascular hyperpermeability, promoting the invasion of pathogens into the bloodstream and causing bloodstream infection. Previous studies have reported that, in the general population, more than 70% of patients with bloodstream infection progress to sepsis, and bloodstream infection is among the most common causes of sepsis [33,34]. Similarly, bacteremia was the leading cause of infection leading to sepsis in our study, followed by pneumonia. When bacteria are present in the bloodstream, some endotoxins, such as bacterial lipopolysaccharide (LPS), and pro-inflammatory cytokines can be released. Flemming et al. demonstrated that tumor necrosis factor-α and LPS can induce VE-Cadherin shedding from the endothelium and increase vascular permeability [21]. This can further impair vascular integrity and cause vascular leakage, allowing fluid and proteins to leak into surrounding tissues and leading to tissue edema and multi-organ dysfunction [35,36]. Our study found a significantly higher baseline VE-Cadherin level in patients with sepsis than in those without sepsis. This may indicate pre-existing vascular hyperpermeability in these sepsis patients and predispose them to the risk of sepsis. Moreover, we observed that VE-Cadherin levels decreased in the first week after LVAD implantation in both groups. Due to impaired heart ejection function in these advanced heart failure patients, tissue hypoperfusion and elevated blood lactate levels are common. One previous study by Yang et al. reported that lactate can induce vascular permeability by disrupting VE-Cadherin [37]. Following LVAD implantation, end-organ and microvascular perfusion can improve, more blood and oxygen can be delivered, and blood lactate levels can decrease. This may explain why VE-Cadherin levels can decrease in the first week after LVAD implantation; improvements in the hemodynamic profile may benefit vascular integrity. It is important to note that, in our cohort, VE-Cadherin and ADAM10 levels were measured only before surgery and during the first four weeks postoperatively. However, the median onset of sepsis occurred at 56 days. Therefore, all biomarker levels reported in this study reflect early measurements taken before the onset of sepsis. While this is crucial for evaluating their predictive value, it does not capture the behavior of these biomarkers at the time when clinical sepsis manifests.

A previous prospective study by Zhang et al. reported that higher levels of soluble VE-Cadherin in sepsis patients were associated with poorer outcomes [19]. Similarly, Yu et al. conducted a prospective study of sepsis patients with and without severe acute kidney injury. They found that shedding of VE-Cadherin is associated with severe acute kidney injury and with more severe organ dysfunction in patients with sepsis. VE-Cadherin was proposed as a valuable biomarker for predicting sepsis outcomes in critically ill patients [18]. However, all these studies focused on VE-Cadherin’s predictive ability for sepsis severity and prognosis. In our study, we examined the predictive performance of VE-Cadherin for post-operative sepsis in LVAD patients using multiple time point values. We found that VE-Cadherin levels from baseline to 4 weeks postoperatively are a reliable biomarker for predicting postoperative sepsis. Notably, the plasma VE-Cadherin level at postoperative 1 week demonstrated the highest predictive accuracy, with an AUC of 0.81 and a specificity of 84.6% for sepsis prediction, outperforming other time points. Therefore, we recommend utilizing the postoperative 1-week VE-Cadherin level as a critical predictor of postoperative sepsis in LVAD patients. Furthermore, we observed a significant positive correlation between ADAM10 and VE-Cadherin exclusively in the sepsis cohort. This is consistent with previous studies, which have found that circulating ADAM10 correlates positively with VE-Cadherin levels in aortic aneurysm and dissection populations; moreover, mechanistic experiments have demonstrated that upregulated ADAM10 increases soluble VE-cadherin levels in cell culture supernatants, whereas ADAM10 inhibition prevents its shedding [38]. Moreover, Burg et al. used a rheumatoid arthritis mouse model and demonstrated that ADAM10 is the principal VE-cadherin “sheddase” [39]. Similarly, Schulz et al. used gain-of-function analyses, inhibitor studies, and RNA interference experiments in human umbilical vein endothelial cells to show that VE-cadherin is specifically cleaved by ADAM10 in its ectodomain, thereby releasing a soluble fragment into the circulation [24]. Consistently, Maretzky et al. reported that ADAM10 is the critical enzyme mediating VE-cadherin shedding in epithelial cells; in ADAM10-deficient mouse embryos, the C-terminal VE-cadherin fragment was not generated [22]. Collectively, these findings highlight ADAM10 as the key enzyme responsible for VE-cadherin shedding and increased vascular permeability in multiple models. We propose that similar mechanisms may operate in the LVAD population, predisposing patients to an elevated risk of sepsis. After incorporating ADAM10 into the VE-Cadherin prediction model, the model’s predictive performance improved across all five time points, further supporting its role in the pathophysiology and risk stratification of postoperative sepsis in LVAD patients. Furthermore, ADAM10 has been implicated in the pathogenesis of Staphylococcus aureus sepsis [27]. S. aureus α-toxin (Hla) not only induces direct cellular injury through pore formation but also activates ADAM10 metalloprotease activity, leading to pathological cleavage of VE-Cadherin on endothelial cells. ADAM10 may be a promising therapeutic target for the prevention and treatment of sepsis [40,41]. Indeed, a recent study by Wang et al. identified a novel ADAM10 inhibitor, “Kaempferol,” as a potential treatment for Staphylococcus aureus infection. Kaempferol can reduce VE-cadherin shedding and maintain vascular integrity, highlighting its potential as a therapeutic strategy to mitigate endothelial dysfunction in sepsis [42]. However, in the current study, our primary aim was to evaluate the efficacy of VE-Cadherin in predicting sepsis in LVAD patients. We examined the expression trajectory of ADAM10 and its correlation with circulating VE-Cadherin, but we did not perform mechanistic experiments. Future studies are warranted to validate whether ADAM10 directly mediates VE-Cadherin shedding in this setting and to test whether ADAM10 inhibition can mitigate sepsis risk in LVAD patients. Unlike the general population, LVAD-supported patients are at increased risk of infection due to altered hemodynamics, impaired immune function, and the presence of an external driveline [7]. LVAD-specific infections, such as driveline infections, occur at rates ranging from 7% to 71% within the first year [43]. Despite being an indispensable therapy for advanced HF, sepsis remains a preventable complication with modifiable risk factors. Based on our findings, we propose that VE-Cadherin may serve as a useful biomarker to identify high-risk patients. For high-risk patients, we recommend close attention to driveline exit-site hygiene, wound care, and measures to strengthen the immune system. For physicians, the timely initiation of more aggressive antibiotic therapy should be considered once infection occurs to prevent the onset or progression of sepsis.

### Study Limitations

There are several limitations in our study. Firstly, although 106 patients were screened, only 50 were ultimately enrolled due to consent availability and predefined exclusion criteria. LVAD recipients constitute a highly selected population, and declining sepsis rates in contemporary practice, driven by advances in LVAD design and postoperative management, further limited the number of sepsis events despite 3.5 years of enrollment. The small number of events reduces statistical power and increases the risk of bias in predictive modeling. Because only nine sepsis cases occurred, multivariable adjustment for all potential confounders was not statistically feasible without violating the event-per-variable assumption, and residual confounding cannot be excluded entirely, even though baseline characteristics were comparable between groups. In addition, the event count was insufficient to yield stable net reclassification improvement (NRI) or integrated discrimination improvement (IDI) estimates, as these metrics require substantially more events to avoid highly imprecise or inflated results. The incremental predictive value of ADAM10 will need to be confirmed in larger, independent datasets. Second, our predictive models were developed and evaluated using the same dataset, and we did not have an independent validation cohort or perform cross-validation. Although multiple plasma samples were collected at several postoperative time points and internal bootstrap resampling was applied to estimate and correct for model optimism, these approaches cannot entirely eliminate the risk of inflated performance metrics when independent data are not available. Therefore, this study should be interpreted as preliminary and hypothesis-generating. External validation in larger, independent cohorts will be required before these biomarkers can be implemented in clinical practice. Thirdly, because circulating VE-Cadherin and ADAM10 were not measured at the time of sepsis onset, we were unable to evaluate how these biomarkers change during active sepsis. A previous study reported that circulating VE-Cadherin was significantly elevated during sepsis and correlated with sepsis severity [18]. In our study; however, the primary aim was to evaluate the utility of perioperative and early postoperative VE-Cadherin and ADAM10 levels in predicting later sepsis. Accordingly, all biomarker trajectories represent pre-event values obtained before surgery and during the first four postoperative weeks. These early postoperative patterns may reflect underlying endothelial vulnerability that predisposes patients to sepsis, supporting their potential predictive value. Still, they do not allow conclusions regarding biomarker behavior immediately before or during septic episodes. This temporal separation limits mechanistic interpretation and prevents assessment of biomarker dynamics during sepsis progression. Moreover, we examined only changes in circulating ADAM10 and VE-cadherin expression; the effect of LVAD on ADAM10 activity remains unclear, and the relationship among ADAM10 activity, expression, and VE-cadherin in LVAD patients warrants further investigation. Fourthly, our study measured circulating levels of ADAM10 and VE-Cadherin and did not directly assess vascular permeability. While these biomarkers are mechanistically linked to vascular permeability in preclinical studies, their causal role in mediating vascular leakage in LVAD patients remains to be confirmed in future studies. Fifthly, biomarker measurements were collected only from preoperative through week 4 postoperatively, and follow-up was limited to 180 days to evaluate early and intermediate sepsis events within the biologically relevant window of early biomarker sampling. As a result, late-onset sepsis occurring more than 180 days after admission was not captured. Future studies with longer follow-up and extended longitudinal biomarker sampling (e.g., 6-month or 1-year measurements) will be needed to assess the prediction of late-onset sepsis. Finally, the prevalence of sepsis in our cohort was relatively low, and most cases occurred at the septic shock stage. Although three sepsis patients developed acute ARDS and required ECMO support, the small number of ARDS events and the fact that all biomarker measurements were obtained before the onset of sepsis precluded evaluation of the relationship between VE-cadherin levels and ARDS development in LVAD patients. For the same reason, we were also unable to examine associations between VE-cadherin levels and different stages or severities of sepsis.

## 4. Materials and Methods

### 4.1. Study Design and Groups

This investigation was conducted at our center from 1 June 2020 to 31 December 2023. The study protocol, execution, and reporting adhered to the Standards for Reporting of Diagnostic Accuracy guidelines. Patients with end-stage heart failure who were scheduled for LVAD implantation were prospectively and consecutively recruited. The exclusion criteria included: age < 18 years; sepsis history; LVAD replacement implantation; developed sepsis in the first month after receiving LVAD implantation; and lost to follow-up visits. Exclusion criteria were not related to illness severity, and no systematic differences were observed between included and excluded patients during screening. The institutional IRB (H-45532) approved the study, and informed consent was obtained from all patients.

### 4.2. Biomarker Measurements

Blood samples anticoagulated with K_2_-EDTA were collected at baseline (one day before surgery) and again at postoperative weeks 1, 2, and 3, and 1 month during ICU or follow-up clinic visits. Samples were centrifuged within one hour of collection at 2500 rpm for 15 min, and the resulting plasma was aliquoted into 1.5 mL tubes and stored at −80 °C for later assays without repeated freeze–thaw cycles. The median storage duration prior to biomarker measurement was 5 months (range: 3–7 months), with no systematic differences between the sepsis and no-sepsis groups. All samples from the same patient underwent a single freeze–thaw cycle and were analyzed within the same enzyme-linked immunosorbent assay (ELISA) batch to minimize inter-assay variability. Plasma VE-Cadherin and ADAM10 concentrations were quantified using commercially available ELISA kits (ADAM10, Cat. No: ab30931, Waltham, MA, USA; VE-cadherin, Cat. No: DCADV0, Minneapolis, MN, USA) following the manufacturers’ protocols. All samples were measured in duplicate, and mean values were used for analysis. Calibration curves were generated using recombinant protein standards provided with each kit. The intra-assay coefficients of variation were 5.6% for VE-cadherin and 3.6% for ADAM10, and the inter-assay coefficients of variation were 4.4% and 4.8%, respectively.

### 4.3. Clinical Endpoints and Definitions

The primary endpoint was the presence of sepsis between post-operative day 30 to post-operative day 180 after LVAD implantation. The secondary endpoint was 1-year mortality after LVAD implantation. To diagnose sepsis, we used the Systemic Inflammatory Response Syndrome (SIRS) criteria as outlined in the International Guidelines for Management of Sepsis and Septic Shock 2021 [44]. Patients were classified as having sepsis if they presented with a documented or strongly suspected infection together with two or more SIRS criteria (temperature > 38 °C or <36 °C, heart rate > 90 beats/min, respiratory rate > 20 breaths/min or PaCO_2_ < 32 mmHg, and white blood cell count > 12,000/mm^3^, <4000/mm^3^, or >10% bands. Two board-certified physicians independently reviewed each case to verify that the infection was clinically significant and met the required criteria. Furthermore, according to the Sepsis-3 criteria, septic shock was defined as (i) requiring vasopressor support to maintain a mean arterial pressure ≥ 65 mmHg and (ii) a serum lactate level > 2 mmol/L in the absence of hypovolemia [45]. The identified infection that leads to sepsis was defined as the source of infection. Moreover, according to the latest consensus statement from the International Society of Heart and Lung Transplantation [46], we further classify infections as either mechanical circulatory support (MCS)-specific or non-MCS-specific. MCS-specific infections are those directly attributable to the device hardware and are not encountered in patients without mechanical circulatory support. Non-MCS-specific infections, although unrelated to the hardware surface, may occur independently and can still impact device performance or patient outcomes [46].

### 4.4. Statistical Analysis

All statistical analyses and graphics were generated using GraphPad Prism 9.5.1 and SAS 9.4. Continuous variables are reported as mean ± SD or median (IQR), whereas categorical variables are reported as percentages, following the conventions used in prior studies [47,48,49]. Between-group comparisons for continuous variables were performed using repeated-measures ANOVA or the Mann–Whitney U test. Categorical variables were evaluated using either the Chi-square test or Fisher’s exact test. For biomarker comparisons across multiple time points between sepsis and no-sepsis groups, two-way analysis of variance (ANOVA) was used with group and time as main factors. Bonferroni-corrected post hoc tests were conducted to assess pairwise differences. A *p*-value of less than 0.05 was considered statistically significant. To evaluate the performance of VE-Cadherin as a biomarker for sepsis, we conducted logistic regression models for each time point. Predictions were compared with actual outcomes, and receiver operating characteristic (ROC) curves and confusion matrices were used to summarize classification performance by displaying true positives, true negatives, false positives, and false negatives. We recorded the area under the ROC curve (AUC), sensitivity, specificity, and corresponding 95% confidence intervals (CIs). Internal validation was performed using 1000 bootstrap resamples with replacement. Logistic regression models predicting sepsis from ADAM10 and VE-Cadherin were refit in each resample, and the AUC was calculated. Optimism-corrected AUCs were obtained by subtracting the mean optimism (bootstrap AUC—original AUC) from the apparent AUC. Validation AUCs were also computed on out-of-sample data across all iterations, and mean values with 95% confidence intervals were reported.

## 5. Conclusions

Our study has shown that plasma VE-Cadherin levels can be a valuable biomarker for predicting sepsis in LVAD patients, with predictive performance further enhanced when combined with circulating ADAM10 levels.

## Figures and Tables

**Table 1:** Demographic and clinical characteristics of sepsis and no-sepsis patients.

Characteristics	No-Sepsis (*N* = 41)	Sepsis (*N* = 9)	*p*-Value
Demography			
Age in years, Median (IQR)	57 (53–65)	54 (44–72)	0.479
Sex, *n* (% male)	30 (73.17%)	6 (75.00%)	1.000
Race			0.550
Caucasian white, *n* (%)	23 (56.10%)	7 (77.78%)	
African American, *n* (%)	17 (41.46%)	2 (22.22%)	
Asian, *n* (%)	1 (2.44%)	0	
Height, m, Median (IQR)	1.73 (1.65–1.83)	1.77 (1.74–1.83)	0.219
Weight, kg, Median (IQR)	84.32 (72.30–96.60)	86.10 (73.50–95.30)	0.830
BMI, kg/m^2^, Median (IQR)	27.98 (23.56–30.75)	27.48 (23.39–30.04)	0.950
BSA, m^2^, Median (IQR)	1.99 (1.79–2.20)	2.07 (2.02–2.17)	0.441
History of smoking, *n* (%)	19 (46.34%)	5 (55.56%)	0.721
History of alcohol abuse, *n* (%)	18 (43.90%)	4 (44.44%)	1.000
History of drug abuse, *n* (%)	5 (12.20%)	2 (22.22%)	0.595
Cancer, *n* (%)	3 (7.69%)	0	1.000
Hypertension, *n* (%)	25 (60.98%)	6 (66.67%)	1.000
Diabetes, *n* (%)	14 (34.15%)	3 (33.33%)	1.000
COPD, *n* (%)	3 (7.32%)	1 (11.11%)	0.560
Peripheral vascular disease, *n* (%)	5 (12.20%)	0	0.570
Cerebral vascular accident, *n* (%)	11 (26.83%)	4 (44.44%)	0.423
Prior cardiac surgeries, *n* (%)	2 (4.88%)	1 (11.11%)	0.456
SBP (mmHg), Median (IQR)	107 (94–116)	106 (99–110)	0.840
DBP (mmHg), Median (IQR)	69 (59–77)	63 (55–77)	0.266
Etiology of heart disease			1.000
Ischemic cardiomyopathy, *n* (%)	22 (53.66%)	5 (55.56%)	
Non-ischemic cardiomyopathy, *n* (%)	19 (47.50%)	4 (44.44%)	
INTERMACS profile, median (IQR)	2.38 (2.00–3.00)	2.29 (1.00–3.00)	0.757
NYHA classification, median (IQR)	3.83 (4.00–4.00)	3.89 (4.00–4.00)	0.769
Echocardiographic parameters			
LviDd in centimeters, *n* (%)	6.70 (6.02–7.17)	6.88 (6.39–6.95)	0.596
LVEF (%)	19.75 (15.70–23.90)	19.77 (15.00–24.60)	1.000
CF-LVADs			1.000
Heartmate III, *n* (%)	32 (64.00%)	7 (77.78%)	
Heart Ware HVAD, *n* (%)	8 (19.51%)	2 (22.22%)	
Others, *n* (%)	1 (2.44%)	0	
LVAD implantation goal			1.000
BTT, *n* (%)	2 (5.00%)	0	
DT, *n* (%)	38 (95.00%)	9 (100.00%)	
Mechanical ventilation after surgery (hrs.), median (IQR)	57 (23–49)	47 (19–68)	0.437
ICU stay after surgery (days), Median (IQR)	15 (9–19)	23 (11–27)	0.285
Length of total hospitalization (days), Median (IQR)	34 (21–40)	46 (27–66)	0.105
One-year mortality, *n* (%)	0	4(44.44%)	* <0.001

Note: Data for continuous variables are expressed as medians (IQR), whereas categorical variables are summarized as frequencies and percentages. Between-group differences were assessed using the non-parametric One-Way ANOVA with Kruskal–Wallis test for continuous data and the Chi-square test for categorical data. Statistical significance was defined as * *p* < 0.05. ANOVA, analysis of variance; BMI, body mass index; BSA, body surface area; BTT, bridge to transplantation; COPD, chronic obstructive pulmonary disease; DBP, diastolic blood pressure; DT, destination therapy; ICU; intensive care unit; INTERMACS, Interagency Registry for Mechanically Assisted Circulatory Support; LVEF, left ventricular ejection fraction; LviDd, left ventricular internal diameter at end-diastole; NYHA, New York Heart Association; SBP, systolic blood pressure.

**Table 2:** Two-way ANOVA with post hoc test for group and time effects on ADAM10 and VE-Cadherin levels.

Marker	Group Effect (*p*)	Time Effect (*p*)	Group × Time Interaction (*p*)
VE-Cadherin	<0.0001 *	0.0031 *	0.7499
ADAM10	<0.0001 *	0.0299 *	0.1156

Note: ADAM10, disintegrin metalloproteinases 10; VE-Cadherin, vascular endothelial-cadherin; * *p* < 0.05 is considered significant.

**Table 3:** VE-Cadherin for predicting sepsis in advanced heart failure patients after LVAD implantation.

Timepoints	AUC (95%CI)	Sensitivity (%)	Specificity (%)	*p* Value
VE-Cadherin				
Baseline	0.75(0.61–0.86)	66.7	82.9	0.010 *
Post operative week 1	0.81(0.68–0.91)	77.8	84.6	0.004 *
Post operative week 2	0.69(0.54–0.82)	87.5	66.7	0.047
Post operative week 3	0.72(0.56–0.84)	87.5	61.1	0.035 *
Post operative week 4	0.77(0.62–0.88)	77.8	71.4	0.003 *
VE Cadherin and ADAM10				
Baseline	0.77 (0.58–0.96)	60.2	85.4	0.010 *
Post operative week 1	0.84 (0.64–0.99)	66.6	88.1	0.001 *
Post operative week 2	0.86 (0.74–0.98)	60.0	87.1	0.010 *
Post operative week 3	0.84 (0.68–0.99)	75.0	87.5	0.008 *
Post operative week 4	0.81 (0.64–0.97)	60.0	84.6	0.005 *

Note: ADAM10, disintegrin metalloproteinases 10; VE-Cadherin, vascular endothelial-cadherin; CI, confidence interval. * *p* < 0.05 is considered significant.

## Data Availability

The original contributions presented in this study are included in the article. Further inquiries can be directed to the corresponding author.

## Abbreviations

The following abbreviations are used in this manuscript:

ADAM10	metalloproteinase domain-containing protein 10
BTT	Bridge to Transplantation
LVAD	Left Ventricular Assist Device
DT	Destination Therapy
BMI	Body Mass Index
LVEF	Left Ventricular Ejection Fraction
LViDd	Left Ventricular Internal Diameter at End-Diastole
ICU	Intensive Care Unit
COPD	Chronic Obstructive Pulmonary Disease
SBP	Systolic Blood Pressure
DBP	Diastolic Blood Pressure
INTERMACS	Interagency Registry for Mechanically Assisted Circulatory Support
NYHA	New York Heart Association
ROC	Receiver Operating Characteristic
AUC	Area Under the Receiver Operating Characteristic
VE-Cadherin	Vascular Endothelial Cadherin
HF	Heart Failure
MCS	Mechanical Circulatory Support
WBC	White Blood Cell
CI	Confidence Interval
eGFR	Estimated Glomerular Filtration Rate
INR	International Normalized Ratio
NRI	Net Reclassification Improvement
IDI	Integrated Discrimination Improvement
SIRS	Systemic Inflammatory Response Syndrome
ARDS	Acute Respiratory Distress Syndrome
ECMO	Extracorporeal Membrane Oxygenation
LPS	Lipopolysaccharide
